# Supplementary figures and images for: Reversing Cardiac Hypertrophy at the Source Using a Cardiac Targeting Peptide Linked to miRNA106a: Targeting Genes That Cause Cardiac Hypertrophy
Source: Pharmaceuticals (Basel). 2022 Jul 15;15(7):871. doi: 10.3390/ph15070871 (PMC9317130; doi:10.3390/ph15070871)

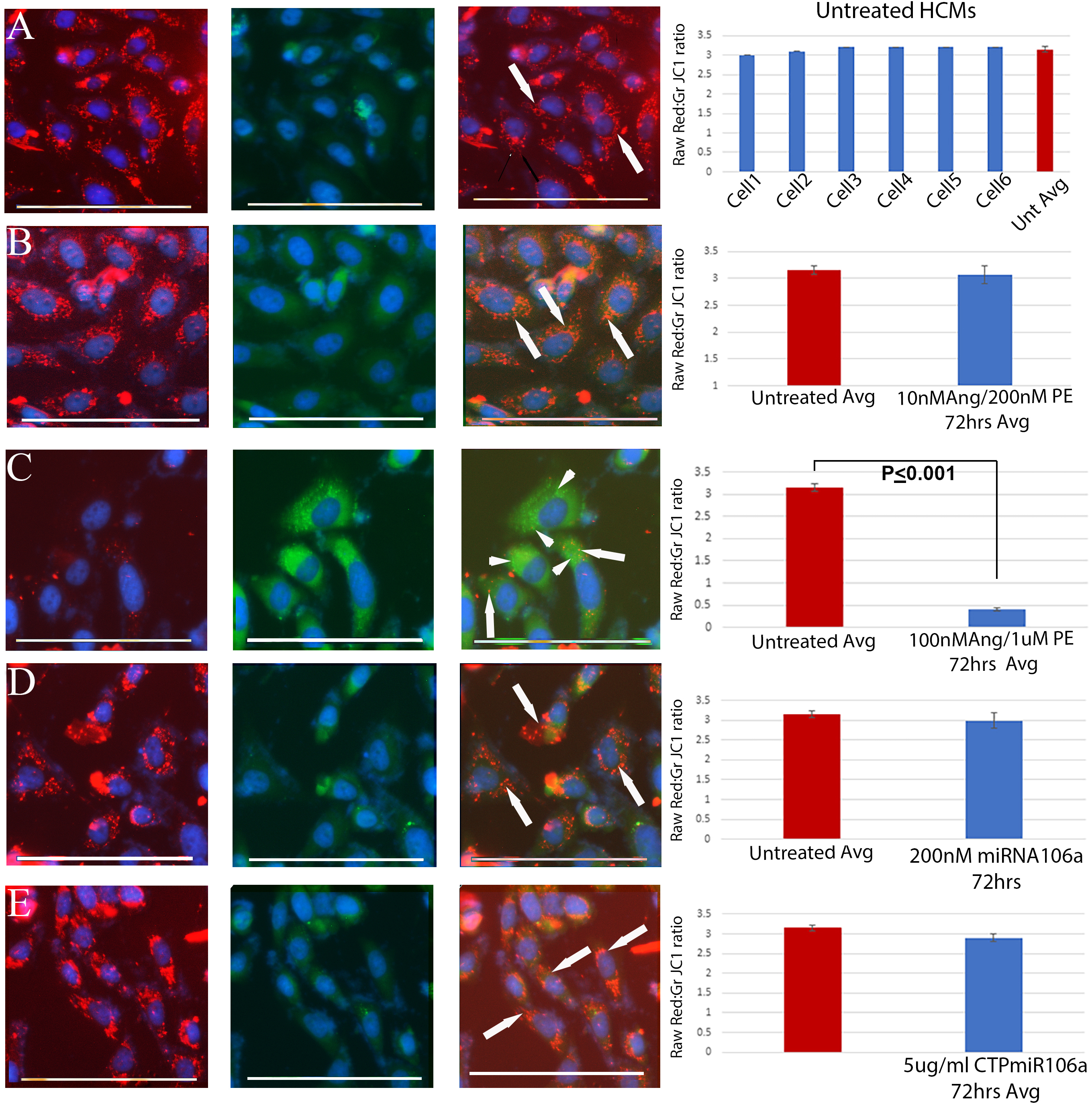

Supplement: Supplementary file 1 [file pharmaceuticals-15-00871-s001.zip › Supplemental Figure S1.jpg]

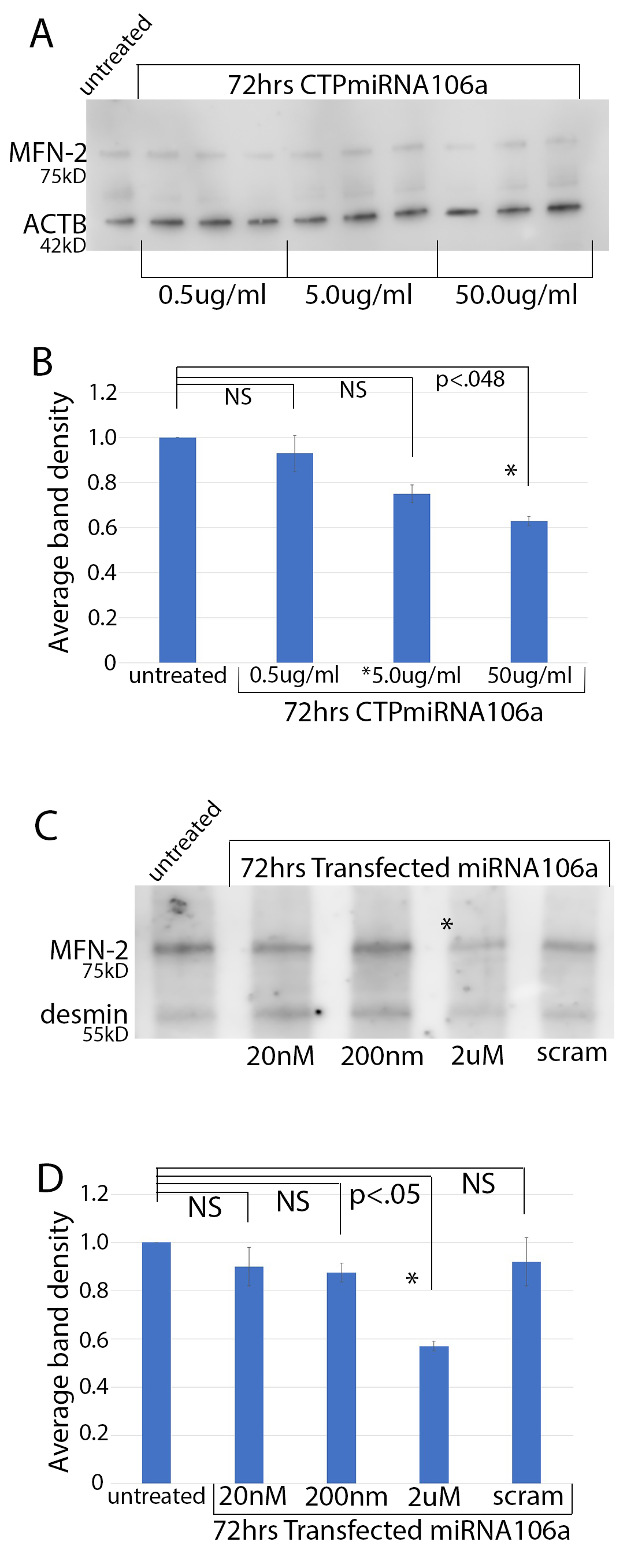

Supplement: Supplementary file 1 [file pharmaceuticals-15-00871-s001.zip › Supplemental Figure S2.jpg]

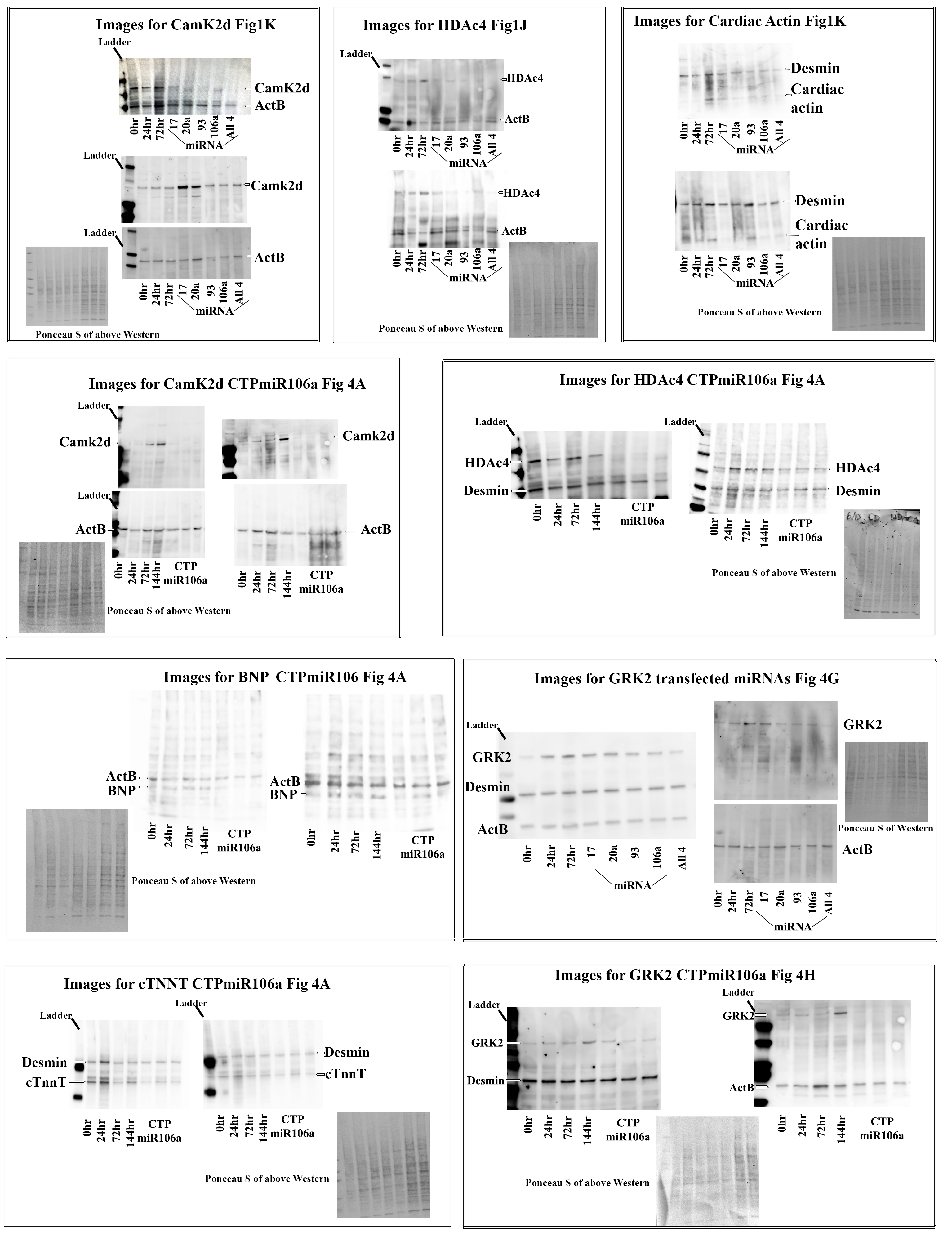

Supplement: Supplementary file 1 [file pharmaceuticals-15-00871-s001.zip › Supplemental Figure S3 Westerns revised.jpg]
